# Photo-Triggered Charge Control Induces Dissociation of Complex Coacervates

**DOI:** 10.3390/polym18060739

**Published:** 2026-03-18

**Authors:** Rei Kakitani, Tomoya Nishimura, Thi Ngan Vu, Chisato Kizaki, Shin-ichi Yusa

**Affiliations:** Department of Applied Chemistry, Graduate School of Engineering, University of Hyogo, 2167 Shosha, Himeji 671-2280, Japan; rei.kaki2001@gmail.com (R.K.); nishitomo200101@gmail.com (T.N.); vungan02091999@gmail.com (T.N.V.); kzk066@gmail.com (C.K.)

**Keywords:** polyampholytes, coacervates, photo-responsive polymers, electrostatic interactions, dissociation behavior

## Abstract

In this study, we designed a statistical polyampholyte bearing cationic quaternary ammonium salts and anionic phosphate groups as pendant functionalities. In addition, small amounts of *o*-nitrobenzyl groups, which generate anionic species upon photoirradiation, were introduced into the pendant chains to prepare a photo-responsive polyampholyte via reversible addition-fragmentation chain transfer radical polymerization. By increasing the feed ratio of the cationic monomer during copolymerization, a polyampholyte with a net positive charge was obtained. Upon photoirradiation of the aqueous solution of this cationic polyampholyte, the fraction of negatively charged groups in the polymer increased, resulting in a decrease in the zeta potential from positive values to around 0 mV. When the photo-responsive cationic polyampholyte was mixed with an anionic polyelectrolyte, poly(2-acrylamido-2-methylpropanesulfonate) (PAMPS), in water, micrometer-sized coacervate droplets were formed via electrostatic interactions. Photoirradiation of the aqueous coacervate system increased the fraction of negative charges in the polyampholyte, thereby weakening the electrostatic interactions with anionic PAMPS and resulting in the dissociation of the coacervates. Overall, this study presents a design guideline for polymeric materials in which interpolymer electrostatic interactions can be controlled by light to induce the disappearance of coacervates.

## 1. Introduction

Coacervates are polymer-rich, water-containing liquid droplets that arise from liquid–liquid phase separation in aqueous media [1,2,3]. Coacervates can be formed either by self-coacervation of a single amphiphilic or ampholytic polymer via liquid–liquid phase separation, or by complex coacervation driven by electrostatic interactions between oppositely charged polymers. This condensed phase, unlike a solid precipitate, remains a fluid phase. Therefore, coacervates have attracted attention as a possible mechanism underlying the formation of membraneless organelles observed in living cells [4,5,6,7]. This fluid, condensed phase enables high loading of guest molecules through selective partitioning and allows molecular diffusion within the droplets, making coacervates attractive for applications such as drug delivery systems (DDSs) [8], microcapsules [9], and enzyme reaction fields [10]. If the formation and dissociation of coacervates can be controlled by external stimuli, their potential applications as functional soft materials will be greatly expanded.

Recently, stimuli-responsive polymers that change their structures and physical properties in response to external stimuli, such as pH, temperature, and light, have attracted considerable attention. Among these stimuli, light is highly tunable in terms of wavelength, irradiation time, and intensity, and can be readily turned on and off [11,12,13]. Furthermore, because no chemical reagents need to be added to the system, the risk of chemical contamination is minimal. For these reasons, light is considered a highly versatile external stimulus, and its applications in DDS [14,15] and photolithography/photopatterning [16] have been extensively explored.

To date, coacervates that undergo formation and dissociation in response to various stimuli, such as pH [17], temperature [18], and redox conditions [19], have been reported. Light-responsive coacervate-related systems have also been explored [20,21,22]; however, their operating principles differ from those of the present study. For example, some systems regulate liquid–liquid phase separation or coacervation through photoisomerization-induced changes in molecular packing, polarity, or thermoresponsive behavior, whereas others use photodeprotection of ionizable groups to initiate polyelectrolyte complex formation. In contrast, the present study focuses on the light-triggered dissociation of a pre-formed polymeric complex coacervate by shifting the net charge balance of one complexing polymer through the photo-generation of additional anionic groups. To the best of our knowledge, examples of this strategy remain relatively limited.

**Figure 1:**
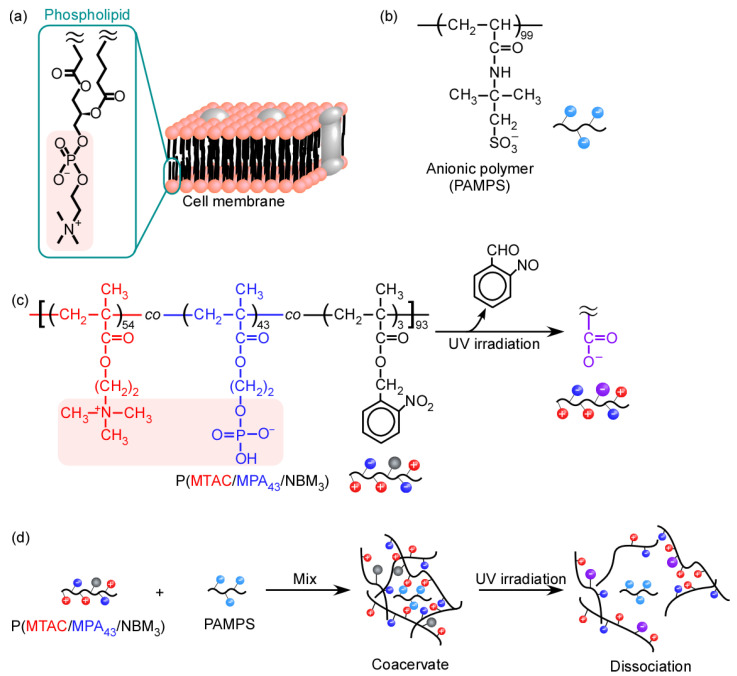
(**a**) Schematic illustration of a cell membrane, (**b**) chemical structure of PAMPS, (**c**) chemical structure of P(MTAC/MPA_43_/NBM_3_), and (**d**) schematic illustration of coacervate formation via electrostatic interactions and its dissociation upon photoirradiation.

A betaine-type zwitterionic polymer bearing both positive and negative charges within the pendant chains is referred to as a polybetaine and exhibits unique hydration behavior characterized by a strongly bound hydration layer. Consequently, nonspecific interactions with proteins and cells are generally suppressed [23,24,25,26]. A representative example is poly(2-methacryloyloxyethyl phosphorylcholine) (PMPC), which bears pendant phosphorylcholine (PC) groups (Figure 1a) that are structurally similar to the hydrophilic moieties on cell membrane surfaces. PMPC has a polyphosphobetaine structure and exhibits excellent biocompatibility with protein antifouling properties [27,28,29]. In addition, polymers bearing choline phosphate (CP) groups, which have a charge configuration opposite to that of PC groups, exhibit markedly different aggregation behavior from their PC analogs. This observation indicates that the composition and arrangement of pendant charges strongly affect polymer-polymer interactions [30]. These results suggest that tuning the charge balance during polymer synthesis provides an effective strategy for controlling coacervate formation. Generally, polyampholytes are prepared via random copolymerization of cationic and anionic monomers. The overall charge balance can be precisely tuned by adjusting the polymer composition. When the overall charge is close to neutral, nonspecific interactions with proteins and cells are suppressed [31,32,33,34].

In the present study, the phosphate-bearing monomer MPA was selected not to reproduce the complete structure of a natural phospholipid, but to introduce a biologically relevant and strongly hydrated phosphate motif into the side chains of a methacrylate-based polyampholyte. In combination with the permanently cationic MTAC unit, this molecular design was inspired by phospholipid headgroup chemistry and provided a useful platform for examining how phosphate-containing pendant groups influence interpolymer electrostatic interactions, coacervate formation, and photo-triggered dissociation. In this study, we designed a polyampholyte by random copolymerization of 2-(methacryloyloxy)ethyl trimethylammonium chloride (MTAC) and 2-methacryloyloxyethyl phosphate (MPA). Furthermore, we prepared a terpolymer (P(MTAC/MPA_43_/NBM_3_)) by incorporating a small amount of *o*-nitrobenzyl methacrylate (NBM), which generates anionic species upon photoirradiation (Figure 1c). All monomers were methacrylate derivatives to minimize differences in comonomer reactivity. Upon photoirradiation, NBM undergoes cleavage of the *o*-nitrobenzyl group from the pendant chain, generating carboxylate anions and photoproducts derived from the *o*-nitrobenzyl moiety, mainly *o*-nitrosobenzaldehyde [35,36,37]. Consequently, photoirradiation shifts the overall charge balance of the polymer toward a more anionic state [38,39,40]. During polymerization, MTAC was used in slight excess relative to MPA, thereby biasing the net charge of the polymer toward a cationic character. Accordingly, before photo-irradiation, P(MTAC/MPA_43_/NBM_3_) was weakly cationic, allowing electrostatic interactions with the anionic polymer. However, photoirradiation increased the anionic character of the polymer, thereby weakening the electrostatic interactions (Figure 1d). By utilizing this photo-induced change in charge state, we designed coacervates based on electrostatic interactions between a cation-biased P(MTAC/MPA_43_/NBM_3_) and anionic poly(2-acrylamido-2-methylpropanesulfonate) (PAMPS). PAMPS bears anionic sulfonate groups in its pendant chains (Figure 1b). This study aimed to fabricate photo-dissociable coacervates. We also assessed how photo-induced changes in pendant charge affect coacervate stability. Such photo-responsive polyampholytes may be applicable to stimuli-responsive soft materials.

## 2. Materials and Methods

### 2.1. Materials

An aqueous solution of 2-(methacryloyloxy)ethyltrimethylammonium chloride (MTAC) was purchased from Sigma-Aldrich (St. Louis, MO, USA). Although the catalog concentration is 80 wt%, the MTAC solution used in this study was confirmed to be 83 wt% in H_2_O by ^1^H NMR spectroscopy. *o*-Nitrobenzyl methacrylate (NBM, ≥95%) was purchased from Polysciences (Warrington, PA, USA). Prior to use, MTAC and NBM were passed through a polymerization inhibitor removal column (Sigma-Aldrich) to remove inhibitors. 2-Methacryloyloxyethyl phosphoryl dimethyl ester (MPDME) was synthesized according to the literature [41]. 2-Methacryloyloxyethyl phosphate (MPA) was kindly provided by Johoku Chemical Industry (Tokyo, Japan) and used as is without further purification received. 4-Cyanopentanoic acid dithiobenzoate (CPD) was synthesized according to the literature [42]. Azobisisobutyronitrile (AIBN, 98.0%) and sodium acetate trihydrate (98.5%) were purchased from Fujifilm Wako Pure Chemical (Osaka, Japan) and used as received. Methanesulfonic acid (>99.0%), dimethyl sulfide (>99.0%), 2-hydroxyethyl methacrylate (HEMA, >95.0%), dimethyl chlorophosphate (DCP, >98.0%) and 2,2,2-trifluoroethanol (TFE) were purchased from Tokyo Chemical Industry (Tokyo, Japan) and used as received. Ion-exchanged water was used as purified water. PAMPS (DP = 99, *M*_n_ = 2.24 × 10^4^ g/mol, *M*_w_/*M*_n_ = 1.17) was prepared via RAFT polymerization and purified by dialysis according to a procedure previously established in our laboratory (Figures S1 and S2).

### 2.2. Measurements

^1^H NMR spectra were recorded on a JEOL (Tokyo, Japan) JNM-ECZ 400 MHz spectrometer. Dynamic light scattering (DLS) measurements were performed using a Malvern (Worcestershire, UK) Zetasizer Pro Blue to determine the hydrodynamic radius (*R*_h_), light scattering intensity (LSI), and zeta potential. In addition to the average *R*_h_ values, intensity-weighted *R*_h_ distributions were obtained from the autocorrelation functions using the instrument software. UV-vis absorption spectra and percentage transmittance (%*T*) were measured using a JASCO (Tokyo, Japan) V-730 UV/VIS spectrophotometer. Gel-permeation chromatography (GPC) of P(MTAC/MPA_43_/NBM_3_) was performed at 40 °C using an acetic acid-based eluent (0.5 M CH_3_COOH, 0.3 M Na_2_SO_4_). The GPC system consisted of a JASCO (Tokyo, Japan) DG-4580 degasser, PU-2080 pump, and RI-2031 Plus RI detector, equipped with two Shodex (Tokyo, Japan) OHpak SB-804 HQ and OHpak SB-806 HQ columns connected in series. GPC of PAMPS was performed at 40 °C using a phosphate buffer-based eluent. The GPC system consisted of a JASCO (Tokyo, Japan) DG-4580 degasser, PU-4580 pump, and RI-4030 RI detector, equipped with Tosoh (Tokyo, Japan) TSKgel α-M column. Photoirradiation was conducted using an Asahi Spectra (Tokyo, Japan) MAX-303 light source equipped with an RLQL80-1 rod lens. The light intensity was measured using a TENMARS ELECTRONICS (Taipei, Taiwan) TM-223 light meter. Microscopic observations were performed using a Keyence (Osaka, Japan) BZ-8000 microscope with a Nikon (Tokyo, Japan) Plan Apo 10× objective (NA = 0.45).

### 2.3. Preparation of P(MTAC/MPDME_43_/NBM_3_)

**Scheme 1:**
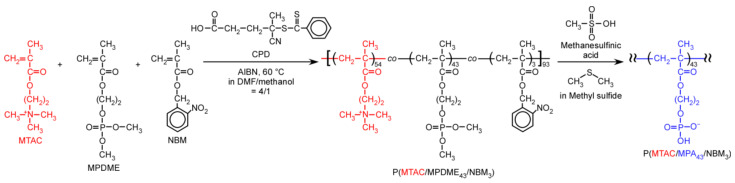
Synthesis of P(MTAC/MPA_43_/NBM_3_).

83 wt% aqueous solution of MTAC (1.87 g, 7.50 mmol as MTAC), MPDME (1.64 g, 6.75 mmol), and NBM (165 mg, 0.75 mmol) were dissolved in 15 mL of *N,N*-dimethylformamide (DMF)/methanol (4/1, *v*/*v*) to give a total monomer concentration of 1.0 M. CPD (42.0 mg, 0.15 mmol) and AIBN (9.84 mg, 60.0 μmol) were then added to the solution to afford a molar ratio of [MTAC]/[MPDME]/[NBM]/[CPD]/[AIBN] = 50/45/5/1/0.4. The solution was degassed with Ar for 30 min and then heated at 60 °C for 20 h (Scheme 1). The monomer conversion was 96.5%, as calculated from the decrease in the vinyl group signals in ^1^H NMR (Figure S3). After polymerization, the reaction mixture was dialyzed against acetone for 1 day and then against deionized water for 2 days using a Spectra/Por (Rancho Dominguez, CA, USA) dialysis membrane (MWCO = 6–8 kDa). The polymer (P(MTAC/MPDME_43_/NBM_3_)) was then recovered by lyophilization (2.33 g, 69.2%). The copolymer composition determined by ^1^H NMR was 54, 43, and 3 mol% for MTAC, MPDME, and NBM, respectively. Table 1 summarizes the theoretical degree of polymerization (DP(theo)), theoretical number-average molecular weight (*M*_n_(theo)), DP (DP(NMR)) and *M*_n_ (*M*_n_(NMR)) estimated by ^1^H NMR, *M*_n_ (*M*_n_(GPC)) estimated by GPC, and molecular weight distribution (*M*_w_/*M*_n_) for P(MTAC/MPDME_43_/NBM_3_).

### 2.4. Preparation of P(MTAC/MPA_43_/NBM_3_)

The MPDME units bearing methoxy-protected phosphate groups in P(MTAC/MPDME_43_/NBM_3_) were deprotected to convert them into phosphate groups [43]. P(MTAC/MPDME_43_/NBM_3_) (*M*_n_(NMR) = 2.08 × 10^4^ g/mol, *M*_w_/*M*_n_ = 1.08, 1.85 g, 0.0889 mmol) was dissolved in dimethyl sulfide (5.40 mL, 70.6 mmol). Methanesulfonic acid (4.58 mL, 70.6 mmol) was added to the solution in an ice bath, and the mixture was stirred at room temperature for 24 h (Scheme 1). After the reaction, the solution was dialyzed against acetone for 1 day and then against deionized water for 2 days using a Spectra/Por (Rancho Dominguez, CA, USA) dialysis membrane (MWCO = 6–8 kDa). The polymer (P(MTAC/MPA_43_/NBM_3_)) was then recovered by lyophilization (1.38 g, 74.7%). The copolymer composition determined by ^1^H NMR was 54, 43, and 3 mol% for MTAC, MPA, and NBM, respectively. The DP(theo), *M*_n_(theo), DP(NMR), *M*_n_(NMR), *M*_n_(GPC), and *M*_w_/*M*_n_ of P(MTAC/MPA_43_/NBM_3_) are summarized in Table 1.

### 2.5. Preparation of P(MTAC/MPA_43_/NBM_3_)/PAMPS Complex

P(MTAC/MPA_43_/NBM_3_) and PAMPS were dissolved in acetate buffer (10 mM, pH 5.0) at polymer concentration (*C*_p_) of 2.0 g/L, and the polymers were mixed in predetermined molar ratios to form an association complex. The mixing ratio was defined as *f*^+^ = [cation]/([cation] + [anion]), based on the cation concentration ([cation]) and anion concentration ([anion]) derived from the polymers in solution. Samples were prepared at various *f*^+^ values. The resulting P(MTAC/MPA_43_/NBM_3_)/PAMPS aqueous solution (3 mL) was placed in a quartz cell and irradiated with light (>250 nm, 35 mW/cm^2^) while being stirred with a magnetic stirrer.

## 3. Results and Discussion

### 3.1. Synthesis of P(MTAC/MPA_43_/NBM_3_)

First, we studied the polymerization behavior of the phosphoric acid-containing methacrylate monomer, MPA. MPA is commercially available; however, it has been reported to typically contain impurities consisting of two vinyl groups linked by a phosphoric acid group [44]. This impurity induces cross-linking reactions during polymerization, leading to gelation (Figure S4). Consequently, using MPA directly in radical polymerization without purification is undesirable in terms of polymerization reproducibility and molecular weight control. Therefore, in this study, copolymerization was performed using MPDME, a monomer bearing protected phosphate groups on the pendant chains, followed by post-polymerization deprotection to convert them into phosphate groups. Specifically, HEMA and DCP were reacted to synthesize the MPDME monomer, in which the phosphate group is protected as a methoxy ester. Following synthesis, the formation of MPDME was confirmed by ^1^H NMR spectroscopy (Figure S5). MPDME contains only one vinyl group, making it less prone to unexpected cross-linking reactions during polymerization. Consequently, MPDME is a suitable precursor monomer for copolymerization. Furthermore, post-polymerization treatment with methanesulfonic acid efficiently deprotects the methoxy group, converting it into the desired MPA unit bearing phosphoric acid groups on the pendant chains.

Using RAFT polymerization, MTAC bearing quaternary ammonium salts as pendant chains, MPDME bearing methoxy-protected phosphate groups, and the photo-responsive NBM were randomly copolymerized to synthesize the precursor polymer P(MTAC/MPDME_43_/NBM_3_). Subsequently, the methoxy protecting groups on the MPDME pendant chains were removed and converted to phosphate groups, yielding the photo-responsive polyampholyte P(MTAC/MPA_43_/NBM_3_). Prior to deprotection, a signal originating from the MPDME methoxy group was observed at 3.5–3.6 ppm in the ^1^H NMR spectrum (Figure S6). This signal disappeared completely after deprotection. Furthermore, a signal was observed at 1.9–2.3 ppm in the ^31^P NMR spectrum prior to deprotection (Figure S7). After deprotection, this signal shifted to 0.7–1.1 ppm, indicating successful deprotection. Following deprotection, ^1^H NMR spectra of P(MTAC/MPA_43_/NBM_3_) were recorded, and the copolymer composition was calculated from the integrals of the methylene proton signals assigned to each monomer unit. Specifically, the integral ratios of the methylene groups at 4.3–4.6 ppm (MTAC), 3.9–4.3 ppm (MPA), and 5.3–5.5 ppm (NBM) were utilized. The results indicated that the composition was 54, 43, and 3 mol% for MTAC, MPA, and NBM, respectively. Furthermore, the molar absorptivity (*ε*) of NBM at its absorption maximum wavelength (*λ*_max_ = 263 nm) in TFE was 5.97 × 10^3^ L mol^−1^ cm^−1^ (Figure S8). Using this ε value, the NBM content was calculated from the absorbance at 263 nm of P(MTAC/MPA_43_/NBM_3_) measured in TFE (Figure S9). The obtained NBM content was 3 mol%, consistent with the composition determined from ^1^H NMR.

GPC measurements were performed on the precursor polymer P(MTAC/MPDME_43_/NBM_3_) and the deprotected polymer P(MTAC/MPA_43_/NBM_3_). The obtained *M*_w_/*M*_n_ values were 1.08 and 1.21, respectively, indicating that both polymers had relatively narrow *M*_w_/*M*_n_ (Figure S10). Furthermore, the *M*_n_(GPC) values before and after deprotection were nearly identical. DP(theo) and *M*_n_(theo) were calculated using the following equations (Equations (1) and (2)):

(1) $$\mathrm{DP}\left(\mathrm{theo}\right)\;=\;\frac{{\left[M\right]}_{0}}{{\left[\mathrm{CTA}\right]}_{0}}\;\times \;\frac{\mathrm{Conversion}\;(\%)}{100}$$

(2) $$M_{n}\left(\mathrm{theo}\right)=\mathrm{DP}\left(\mathrm{theo}\right)\;\times \;M_{M}+{\;M}_{\mathrm{CTA}}$$

where [M]_0_ denotes the initial concentration of all monomers, [CTA]_0_ denotes the initial concentration of CTA, MM denotes the average molar mass of the repeat unit based on the copolymer composition, and MCTA denotes the molecular mass of CTA. *M*_n_(GPC) (1.41 × 10^4^ g/mol) obtained by GPC was smaller than *M*_n_(theo) (2.16 × 10^4^ g/mol) calculated from Equation (2). This discrepancy arises because the standard poly(2-vinylpyridine) used for GPC calibration and the target polymer differ in pendant-chain structure and hydrodynamic sizes, leading to different hydrodynamic volumes in solution.

### 3.2. Characterization of P(MTAC/MPA_43_/NBM_3_)

The phosphate groups on the MPA unit pendant chains in P(MTAC/MPA_43_/NBM_3_) possess two protons that dissociate sequentially in response to pH. Therefore, pH titration was performed to evaluate the apparent p*K*_a_ of P(MTAC/MPA_43_/NBM_3_). The p*K*_a_ was calculated using the half-neutralization method. Specifically, P(MTAC/MPA_43_/NBM_3_) was dissolved in 0.1 M HCl aqueous solution at *C*_p_ = 1.0 g/L, and 0.1 M NaOH aqueous solution was added dropwise under stirring. The apparent p*K*_a_ values (p*K*_a1_ and p*K*_a2_) were determined from the half-neutralization points. The first derivative (dpH/d*V*) was calculated from the obtained pH data and plotted against the drop volume (*V*). The maximum value of dpH/d*V* was taken as an indicator of the equivalence point (Figure S11a). At the start of titration, HCl is present in excess. Therefore, the volume required to neutralize the excess HCl is defined as *V*_1_. Furthermore, the volume at the first equivalence point is defined as *V*_2_, and the volume at the second equivalence point is defined as *V*_3_. *V*_2_ and *V*_3_ are the equivalence points corresponding to the first and second stages of neutralization of the phosphate group, respectively. *V*_pKa1_ = (*V*_1_ + *V*_2_)/2 and *V*_pKa2_ = (*V*_2_ + *V*_3_)/2, with the pH values at *V*_pKa1_ and *V*_pKa2_ defined as p*K*_a1_ and p*K*_a2_, respectively. The results yielded p*K*_a1_ = 2.08 and p*K*_a2_ = 6.90. These values are close to the literature values (p*K*_a1_ = 2.29 and p*K*_a2_ = 6.40) [45]. These results indicate that the phosphate groups in P(MTAC/MPA_43_/NBM_3_) pendant chains exhibit two-step (diprotic) dissociation behavior in response to pH changes. Note that in polymer electrolytes, the dissociation equilibrium may be altered by neighboring charges; therefore, the p*K*_a_ values obtained in this study should be regarded as apparent values.

To evaluate the charge state of P(MTAC/MPA_43_/NBM_3_), the polymer was dissolved in 0.01 M NaCl aqueous solution at *C*_p_ = 1.0 g/L, and the zeta potential was measured at each pH (Figure S11b). Under these conditions, at pH ≤ 2, the phosphate groups in the MPA unit pendant chains are predominantly protonated. At pH 4–5, the first stage of deprotonation of the phosphate group proceeds, and the MPA unit exists primarily as a monovalent anion. Furthermore, at pH ≥ 8, the second stage of deprotonation proceeds, and the proportion of MPA units existing as divalent anions increases. From the pH dependence of the zeta potential, the pH values corresponding to the first and second half-dissociation points were estimated to be 2.93 and 6.56, respectively. The apparent p*K*_a_ values estimated from the zeta potential-pH profile were in reasonable agreement with those obtained from pH titration. Based on these results, at pH 5, the first stage of the two-step dissociation of the phosphate group predominantly occurs, and the pendant-chain phosphate group exists primarily as a monovalent anion. Conversely, the MTAC unit possesses a quaternary ammonium salt in its pendant chain, maintaining a single positive charge independently of pH. Based on these considerations, the association and photo-response behaviors of P(MTAC/MPA_43_/NBM_3_) in aqueous solution were evaluated in acetic acid/sodium acetate buffer (0.01 M, pH 5.0).

**Figure 2:**
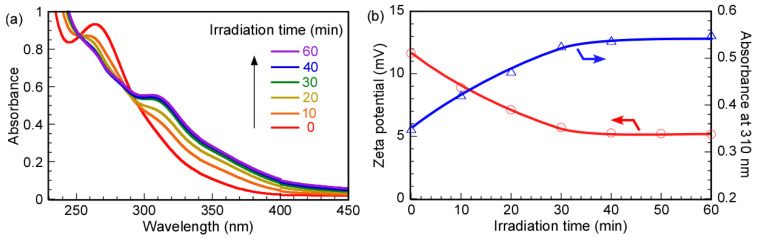
(**a**) UV-vis absorption spectra of P(MTAC/MPA_43_/NBM_3_) in acetate buffer at pH 5.0 and (**b**) zeta potential and absorbance at 310 nm of P(MTAC/MPA_43_/NBM_3_) in acetate buffer at pH 5.0 as a function of photoirradiation time. The arrows in panel (**b**) indicate the corresponding y-axes for the zeta potential and absorbance at 310 nm.

The decomposition behavior of NBM in P(MTAC/MPA_43_/NBM_3_) in a pH 5.0 buffer solution was evaluated by UV-vis absorption spectroscopy upon irradiation with light at wavelengths ≥ 250 nm for 1 h (Figure 2a). The absorbance at 263 nm, originating from NBM, decreased with increasing irradiation time. In contrast, the absorbance at 310 nm, mainly attributed to photoproducts derived from *o*-nitrobenzyl cleavage, including *o*-nitrosobenzaldehyde, increased [35,36,37]. This suggests that photoirradiation cleaves the *o*-nitrobenzyl group from the NBM pendant chains and generates carboxylate (methacrylate) anions on the polymer. It should be noted that *o*-nitrosobenzaldehyde is a reactive intermediate and may undergo secondary reactions. However, the present polymer/coacervate system does not contain primary amine or thiol groups, which are typical reactive partners reported for this species [36,37]. Therefore, although minor secondary reactions cannot be completely excluded, the dominant effect under the present conditions is considered to be the photo-induced increase in anionic groups on the polymer. The p*K*_a_ of methacrylic acid is reported to be 4.66 [46,47]. Therefore, in aqueous solutions at pH 5.0, the newly generated pendant carboxyl groups are expected to be predominantly deprotonated. Indeed, the zeta potential of P(MTAC/MPA_43_/NBM_3_) in acetic acid buffer (pH 5.0) was 11.6 mV prior to irradiation, and decreased to 5.2 mV after 1 h of photoirradiation due to the generation of methacrylate anions (Figure 2b). Under the same conditions, a pH 5.0 acetic acid buffer solution of P(MTAC/MPA_43_/NBM_3_) was irradiated with light for 1 h, and changes in the hydrodynamic radius (*R*_h_) and light scattering intensity (LSI) were evaluated by DLS (Figure S12). The results showed that during the 1 h irradiation, *R*_h_ ranged from 6.1 to 7.7 nm and LSI ranged from 0.17 to 0.19 Mcps, with no significant changes observed in either parameter. Therefore, under these conditions, although photoirradiation generated anions from the NBM units and decreased the zeta potential, no substantial changes were observed in *R*_h_ or LSI. These results suggest that the association state is relatively unaffected by photoirradiation.

### 3.3. Formation of Coacervates

**Figure 3:**
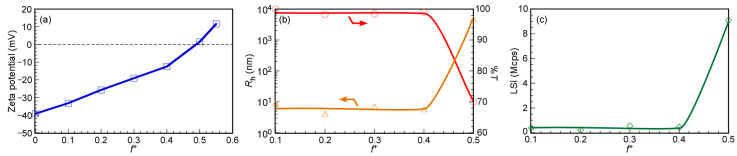
(**a**) Zeta potential, (**b**) hydrodynamic radius (*R*_h_, orange triangle) and percentage transmittance (%*T*, red circle), and (**c**) light scattering intensity (LSI) of P(MTAC/MPA_43_/NBM_3_)/PAMPS mixtures in acetate buffer at pH 5.0 as a function of cationic charge fraction (*f*^+^ = [cation]/([cation] + [anion])). The arrows in panel (**b**) indicate the corresponding y-axes for the *R*_h_ and %*T*.

P(MTAC/MPA_43_/NBM_3_) contains a higher fraction of cationic MTAC units (54 mol%) than anionic MPA units (43 mol%). Consequently, in a pH 5.0 buffer solution, the zeta potential was positive (11.6 mV) prior to photoirradiation. Accordingly, when anionic polyelectrolyte PAMPS was added to the solution, association complexes were expected to form through electrostatic interactions. This study evaluated the effect of the mixing ratio between P(MTAC/MPA_43_/NBM_3_) and PAMPS on their association behavior. The mixing ratio (*f*^+^) was defined based on the concentrations of cationic and anionic charges in solution as *f*^+^ = [cation]/([cation] + [anion]). Here, *f*^+^ = 0 corresponds to PAMPS alone, whereas *f*^+^ = 0.55 corresponds to P(MTAC/MPA_43_/NBM_3_) alone. For each *f*^+^ condition, the zeta potential, *R*_h_, LSI, and percentage transmittance at 700 nm (%*T*) were measured. As *f*^+^ increased, the zeta potential increased. At *f*^+^ = 0.5, where the net charge was nearly neutralized, the zeta potential was 1.5 mV, close to 0 mV (Figure 3a). Within the range 0.1 ≤ *f*^+^ ≤ 0.4, *R*_h_ remained nearly constant at 4.1–8.9 nm (Figure 3b). In this range, electrostatic repulsion was expected to dominate because the net charge was biased toward the anionic side, and no significant polymer–polymer association was observed. In contrast, at *f*^+^ = 0.5, near-neutral charge conditions enabled associative electrostatic interactions, leading to polymer-polymer association and an increase in *R*_h_ to 4.8 µm. The large particle size is consistent with the formation of micrometer-sized coacervate droplets. Furthermore, LSI and %*T* were evaluated for 0.1 ≤ *f*^+^ ≤ 0.5. For 0.1 ≤ *f*^+^ ≤ 0.4, LSI ranged from 0.21 to 0.57 Mcps and %*T* from 98.3 to 99.9%, with no significant changes observed in either parameter (Figure 3c). In contrast, at *f*^+^ = 0.5, LSI increased to 9.1 Mcps and %*T* decreased to 70.0%. These changes support the formation of coacervate droplets via electrostatic interactions under near-charge-neutral conditions. Within the examined composition range, these results identify *f*^+^ = 0.5 as a representative near-charge-neutral condition at which coacervate formation was most clearly observed. A more detailed determination of the phase boundary using finer *f*^+^ intervals would be valuable and is an important subject for future investigation.

### 3.4. Photoresponsive Coacervates

P(MTAC/MPA_43_/NBM_3_) and PAMPS were mixed to achieve *f*^+^ = 0.5 in acetic acid buffer, and the UV-vis absorption spectrum of the resulting coacervate dispersion was recorded (Figure S13). The spectrum obtained immediately after coacervate formation was essentially identical to that measured after 1 h of stirring. These results indicate that under these conditions, the coacervates remained stable without noticeable aggregation for at least 1 h. In contrast, upon irradiation of the coacervate solution with light at wavelengths ≥ 250 nm for 1 h, the absorbance at 263 nm, originating from NBM, decreased. Concurrently, the absorbance at 310 nm, attributed to the photoproduct *o*-nitrosobenzaldehyde, increased (Figure S13). These results confirm that the photolysis of the NBM pendant chains proceeds upon irradiation even in the coacervate state. As in the homogeneous polymer solution, the increase in the 310 nm band is interpreted mainly as the formation of photoproducts derived from *o*-nitrobenzyl cleavage, including *o*-nitrosobenzaldehyde [35,36,37]. Because neither P(MTAC/MPA_43_/NBM_3_) nor PAMPS contains primary amine or thiol groups, extensive covalent trapping of this reactive photoproduct is not expected to be a dominant process in the present system. Nevertheless, minor secondary reactions cannot be completely excluded and should be considered when interpreting the UV-vis spectral changes.

**Figure 4:**
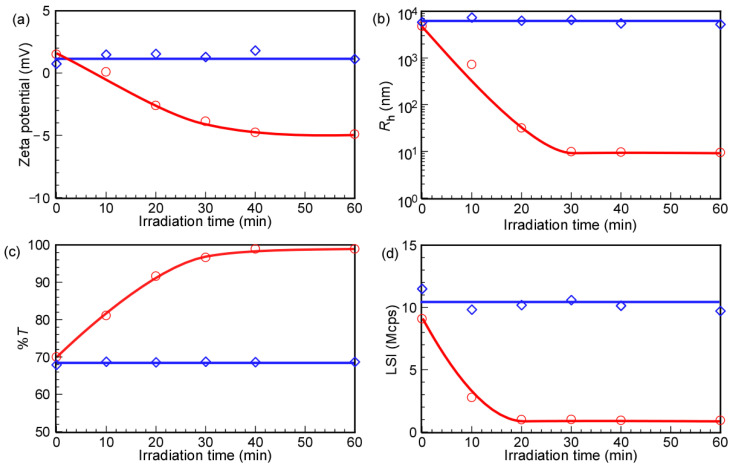
Time-dependent changes in (**a**) zeta potential, (**b**) average hydrodynamic radius (*R*_h_), (**c**) percentage transmittance (%*T*), and (**d**) light scattering intensity (LSI) of P(MTAC/MPA_43_/NBM_3_)/PAMPS in acetate buffer (pH 5.0) at *C*_p_ = 2.0 g/L with UV irradiation (red circle) and without UV irradiation (blue diamond).

**Figure 5:**
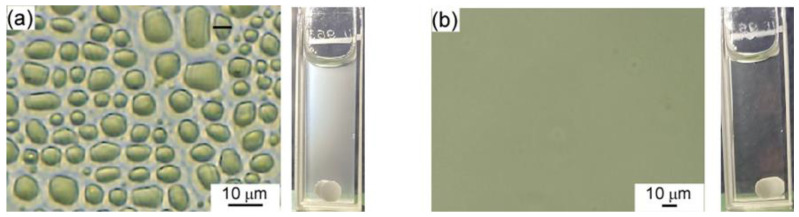
Photographs of P(MTAC/MPA_43_/NBM_3_)/PAMPS mixture at *f*^+^ = 0.5 in acetate buffer at pH 5.0 (**a**) before and (**b**) after photo-irradiation for 1 h.

P(MTAC/MPA_43_/NBM_3_) and PAMPS were mixed to achieve *f*^+^ = 0.5, which was selected as a representative coacervate condition because it exhibited near-charge-neutrality and the most pronounced coacervate characteristics within the examined composition range. The resulting dispersion was then irradiated with light for 1 h, and the irradiation-induced changes in the association behavior were evaluated by monitoring the zeta potential, *R*_h_, %*T* at 700 nm, and LSI (Figure 4). Upon photoirradiation, the zeta potential shifted in the negative direction from 1.5 mV to −4.9 mV. The average *R*_h_ decreased significantly from 4.8 µm to 9.5 nm. %*T* increased from 70.0% to 98.9%. In addition, LSI decreased by approximately one order of magnitude, from 9.1 Mcps to 0.92 Mcps. The intensity-weighted *R*_h_ distribution after 1 h of photoirradiation (Figure S14) shows that the remaining scattering species are centered in the nanometer range, which is consistent with the large decrease in the average *R*_h_. These results support the conclusion that the micrometer-sized coacervate droplets dissociated upon photoirradiation. At the same time, it should be noted that the present experiments do not rigorously establish irreversibility of this process. Although photo-triggered dissociation was clearly observed under the present conditions, re-association experiments, reversal tests, and long-term post-irradiation stability measurements were not performed in this study. Therefore, the present results demonstrate photo-induced dissociation rather than irreversible breakdown in a strict sense. As a control experiment, under identical conditions without photoirradiation, the zeta potential, *R*_h_, %*T*, and LSI remained virtually unchanged. Therefore, under these conditions, the coacervates did not spontaneously dissociate. Furthermore, optical microscopy observations were performed before and after photoirradiation following coacervate formation (Figure 5). Before photoirradiation, micrometer-sized coacervate droplets were clearly observed. To quantify the microscopy results, the droplet radii were measured by image analysis (Figure S15). The average droplet radius obtained from this analysis was 3.23 ± 0.83 μm. In contrast, after photoirradiation, no micrometer-sized droplets were detected under the same observation and analysis conditions. These results quantitatively support the dissociation of the coacervate droplets upon photoirradiation. These results suggest that photoirradiation generates anions from the NBM pendant chains, shifting the net charge of the polymer toward a more anionic state. As a result, the electrostatically driven coacervates dissociate. Unlike previously reported light-responsive phase-separated or polyelectrolyte complex systems that mainly rely on photoisomerization or photoinduced complexation, the present system dissociates a pre-formed polymeric complex coacervate through photo-triggered modulation of the charge balance of a statistical polyampholyte. Because the coacervate was prepared under near-charge-neutral conditions (*f*^+^ = 0.5), even a relatively small increase in the anionic fraction generated from the 3 mol% NBM units is expected to shift the charge balance sufficiently to destabilize the coacervate. A more detailed kinetic analysis of the photocleavage conversion in the coacervate state will be an important subject for future study.

## 4. Conclusions

In this study, we synthesized a photoresponsive polyampholyte, P(MTAC/MPA_43_/NBM_3_), via RAFT polymerization of cationic monomer MTAC, phosphate-containing monomer MPA, and NBM, which generates anions upon photo-irradiation. The resulting polymer exhibited a well-controlled molecular structure, and successful deprotection was confirmed by ^1^H and ^31^P NMR spectroscopy and GPC. Upon photoirradiation in acetate buffer at pH 5.0, the absorbance attributed to NBM decreased, whereas a band mainly attributed to photoproducts derived from *o*-nitrobenzyl cleavage, including *o*-nitrosobenzaldehyde, increased, indicating cleavage of the NBM pendant groups and the concomitant generation of anionic groups. Furthermore, within the examined composition range, mixing P(MTAC/MPA_43_/NBM_3_) with the anionic polymer PAMPS gave the largest *R*_h_ and highest LSI at *f*^+^ = 0.5, where the net charge was nearly neutralized, indicating that this condition was the most suitable for observing coacervate formation in the present study. Upon photoirradiation of the coacervates, *R*_h_, LSI, and zeta potential decreased, whereas %*T* increased. Optical microscopy further showed that the coacervate droplets observed before irradiation disappeared after irradiation, supporting photo-induced dissociation of the coacervates. These results demonstrate that P(MTAC/MPA_43_/NBM_3_) is a photoresponsive polymer whose charge balance can be altered by photoirradiation, thereby enabling control over coacervate dissociation. However, the reversibility of the dissociation process was not examined in the present study, and thus the current data do not constitute a rigorous demonstration of irreversibility.

## Figures and Tables

**Table 1:** Molecular characteristics of the copolymers.

Polymer	DP (Theo)	*M*_n_(Theo) (g/mol)	DP (NMR)	*M*_n_(NMR) (g/mol)	*M*_n_(GPC) (g/mol)	*M*_w_/*M*_n_
P(MTAC/MPDME_43_/NBM_3_)	96	2.16 × 10^4^	93	2.08 × 10^4^	1.41 × 10^4^	1.08
P(MTAC/MPA_43_/NBM_3_)	-	-	93	1.97 × 10^4^	1.25 × 10^4^	1.21

## Data Availability

The original contributions presented in the study are included in the article; further inquiries can be directed to the corresponding author.

## Supplementary Materials

The following supporting information can be downloaded at: https://www.mdpi.com/article/10.3390/polym18060739/s1, Figure S1. ^1^H NMR spectrum of PAMPS in D_2_O with peak assignments and integral intensity values; Figure S2. GPC elution curve of PAMPS using phosphate buffer as the eluent detected by a refractive index (RI) detector; Figure S3. ^1^H NMR spectra for P(MTAC/MPDME_43_/NBM_3_): (a) before polymerization in DMSO-*d*_6_, (b) after polymerization in D_2_O, and (c) after purification in D_2_O with peak assignments and integral intensity values; Figure S4. Representative photograph of PMPA gel after polymerization; Figure S5. ^1^H NMR spectrum of MPDME in CDCl_3_ with peak assignments and integral intensity values; Figure S6. ^1^H NMR spectra of (a) P(MTAC/MPDME_43_/NBM_3_) and (b) P(MTAC/MPA_43_/NBM_3_) in D_2_O with peak assignments and integral intensity values; Figure S7. ^31^P NMR spectra of (a) P(MTAC/MPDME_43_/NBM_3_) and (b) P(MTAC/MPA_43_/NBM_3_) in D_2_O; Figure S8. (a) UV-vis absorption spectra of NBM in 2,2,2-trifluoroethanol (TFE) at various concentrations. (b) Absorbance at 263 nm as a function of NBM concentration ([NBM]) in TFE; Figure S9. UV-vis absorption spectrum of P(MTAC/MPA_43_/NBM_3_) in 2,2,2-trifluoroethanol (TFE) at *C*_p_ = 1.0 g/L; Figure S10. Gel-permeation chromatography (GPC) elution curves of precursor P(MTAC/MPDME_43_/NBM_3_) (**––**) and deprotected P(MTAC/MPA_43_/NBM_3_) (----) using acetic acid as the eluent; Figure S11. (a) pH (**◯**) and dpH/d*V* (**□**) of an aqueous P(MTAC/MPA_43_/NBM_3_) solution as a function of the volume of added NaOH solution (*V*_NaOH_). (b) Zeta potential of P(MTAC/MPA_43_/NBM_3_) as a function of pH in 0.01 M NaCl aqueous solution.; Figure S12. (a) Hydrodynamic radius (*R*_h_) and (b) light scattering intensity (LSI) of P(MTAC/MPA_43_/NBM_3_) at *C*_p_ = 1.0 g/L in acetate buffer at pH 5.0 as a function of photo-irradiation time; Figure S13. UV-vis absorption spectra of P(MTAC/MPA_43_/NBM_3_)/PAMPS at *f*^+^ = 0.5 in acetate buffer at pH 5.0 measured immediately after mixing (**―**), after stirring for 1 h (**— —**), and after irradiation for 1 h (**∙−∙−**); Figure S14. Intensity-weighted hydrodynamic radius (*R*_h_) distributions of P(MTAC/MPA_43_/NBM_3_)/PAMPS at *f*^+^ = 0.5 in acetate buffer at pH 5.0 after photoirradiation for 1 h.; Figure S15. Statistical image analysis of the optical microscopy data for P(MTAC/MPA_43_/NBM_3_)/PAMPS at *f*^+^ = 0.5 in acetate buffer at pH 5.0. Distribution of droplet radius before photoirradiation.
