# Supplementary material for: Photo-Triggered Charge Control Induces Dissociation of Complex Coacervates
Source: Polymers (Basel). 2026 Mar 18;18(6):739. doi: 10.3390/polym18060739 (PMC13030142; doi:10.3390/polym18060739)
Supplement: Supplementary file 1 [file polymers-18-00739-s001.zip › polymers-4182429-supplementary.pdf]

Supporting Information

Photo-Triggered Charge Control Induces Dissociation of Complex Coacervates

Rei Kakitani, Tomoya Nishimura, Thi Ngan Vu, Chisato Kizaki, and Shin-ichi Yusa*

Department of Applied Chemistry, Graduate School of Engineering, University of Hyogo, 2167 Shosha, Himeji, Hyogo 671-2280, Japan

* Correspondence: yusa@eng.u-hyogo.ac.jp

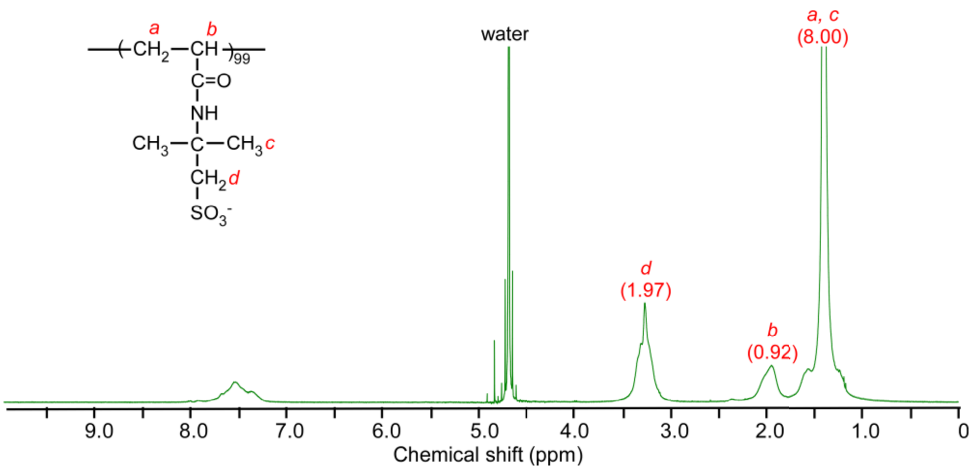

Figure S1. ^{1}H NMR spectrum of PAMPS in D_2O with peak assignments and integral intensity values.

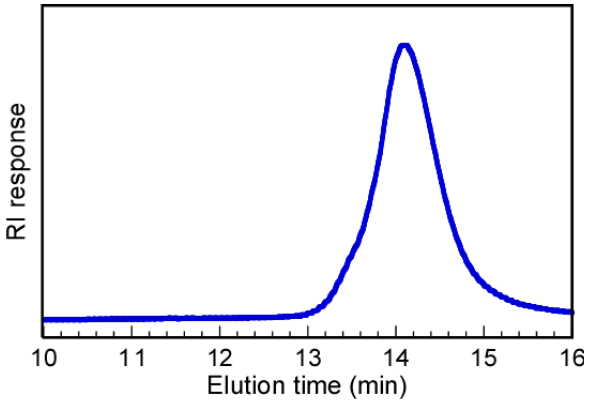

Figure S2. GPC elution curve of PAMPS using phosphate buffer as the eluent, detected by a refractive index (RI) detector.

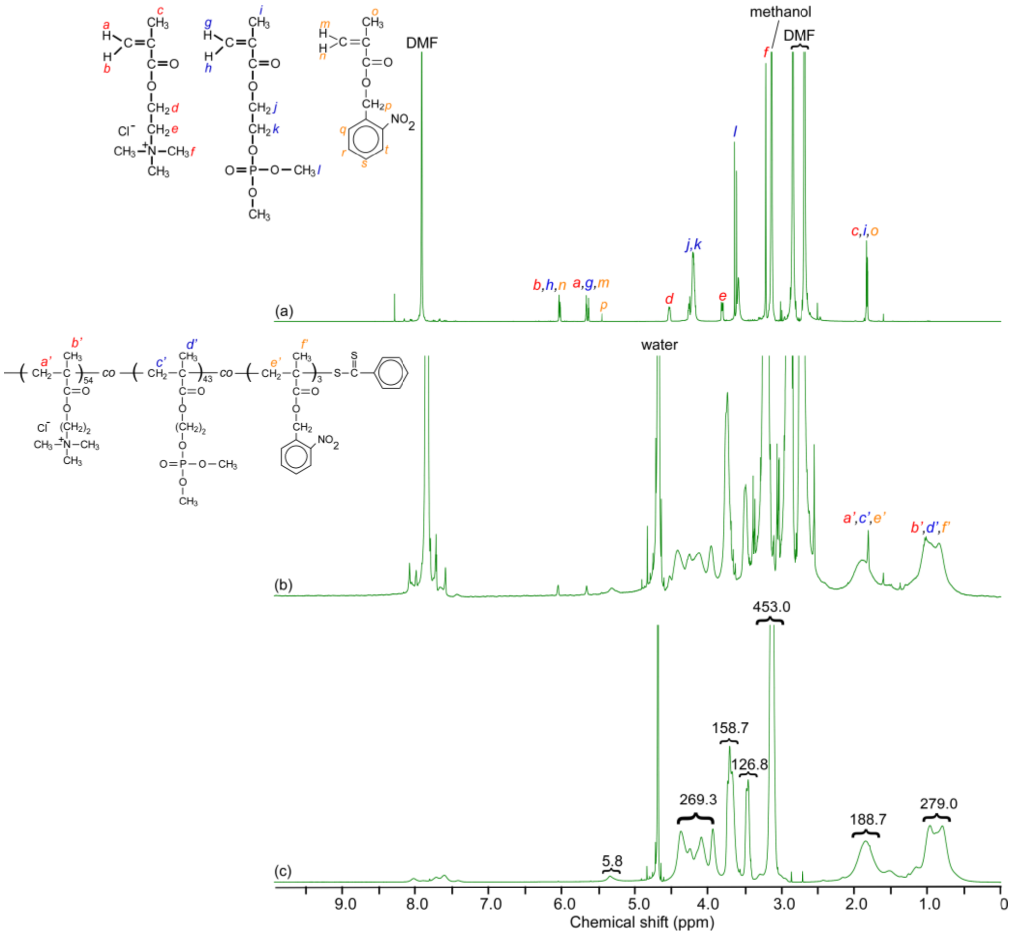

Figure S3. ^{1}H NMR spectra for P(MTAC/MPDME$_{43}$/NBM$_{3}$): (a) before polymerization in DMSO-d_6, (b) after polymerization in D_2O, and (c) after purification in D_2O with peak assignments and integral intensity values.

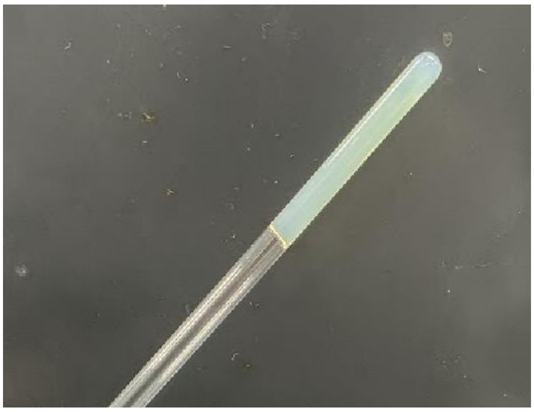

Figure S4. Representative photograph of PMPA gel after polymerization.

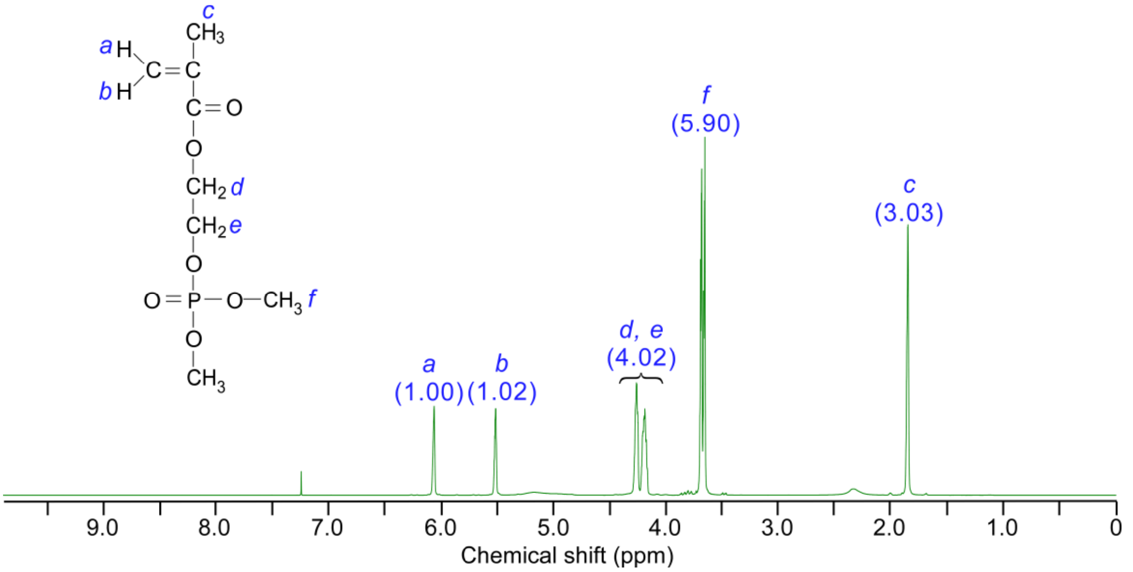

Figure S5. ^{1}H NMR spectrum of MPDME in $CDCl_3$ with peak assignments and integral intensity values.

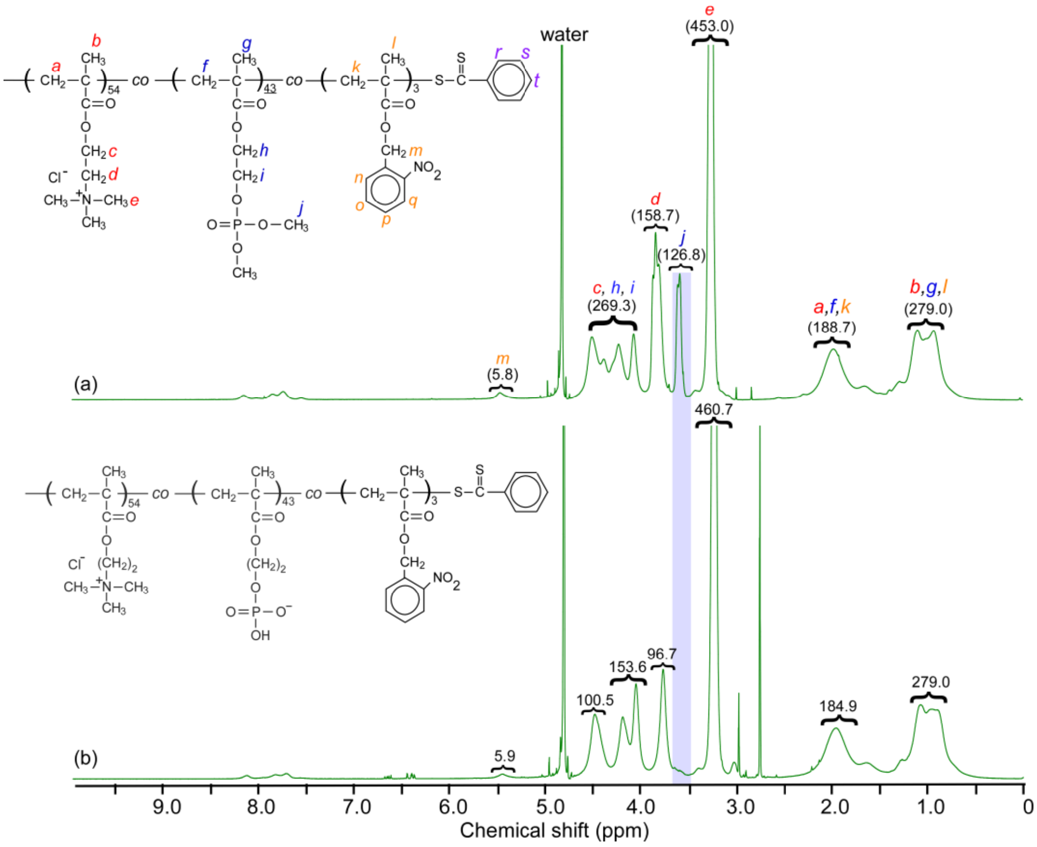

Figure S6. ^{1}H NMR spectra of (a) P(MTAC/MPDME43/NBM3) and (b) P(MTAC/MPA43/NBM3) in D_2O with peak assignments and integral intensity values.

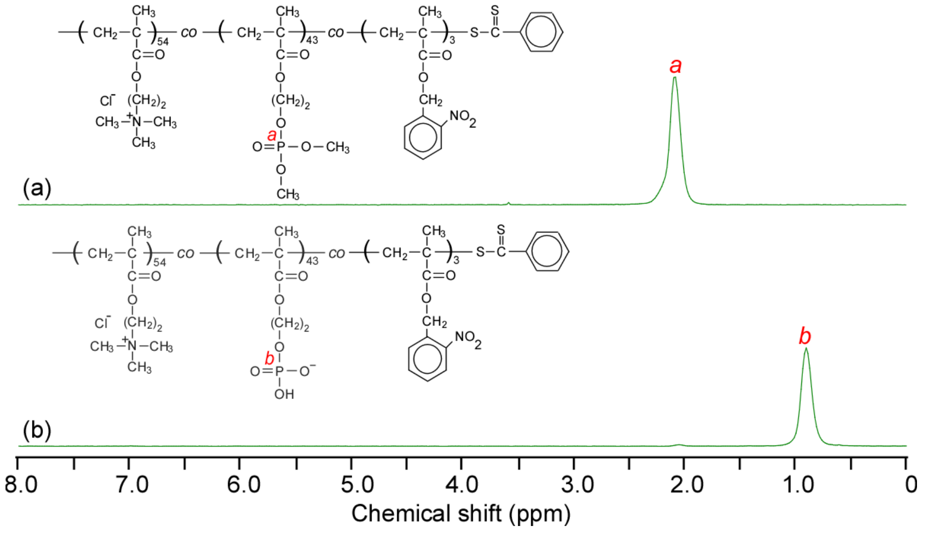

Figure S7. ^{31}P NMR spectra of (a) P(MTAC/MPDME43/NBM3) and (b) P(MTAC/MPA43/NBM3) in D_2O.

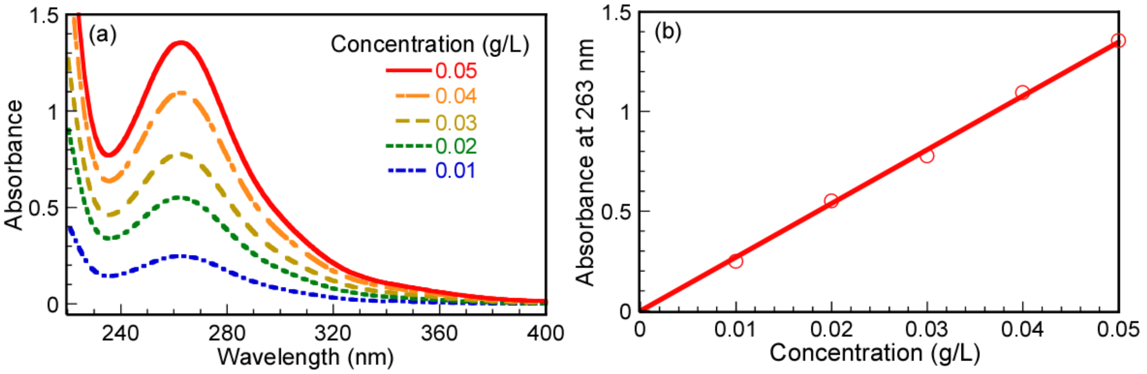

Figure S8. (a) UV-vis absorption spectra of NBM in 2,2,2-trifluoroethanol (TFE) at various concentrations. (b) Absorbance at 263 nm as a function of NBM concentration ([NBM]) in TFE.

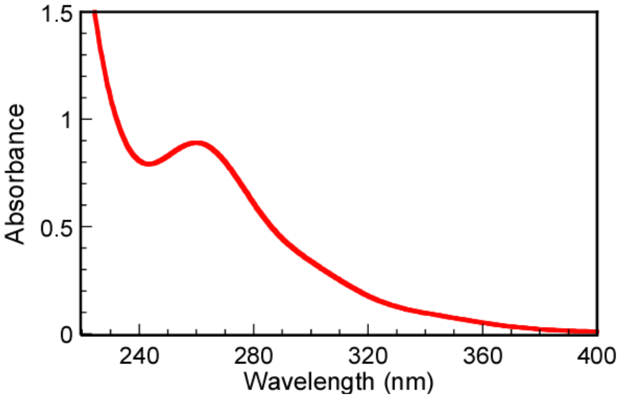

Figure S9. UV-vis absorption spectrum of P(MTAC/MPA$_{43}$/NBM$_3$) in 2,2,2-trifluoroethanol (TFE) at C_p = 1.0 g/L.

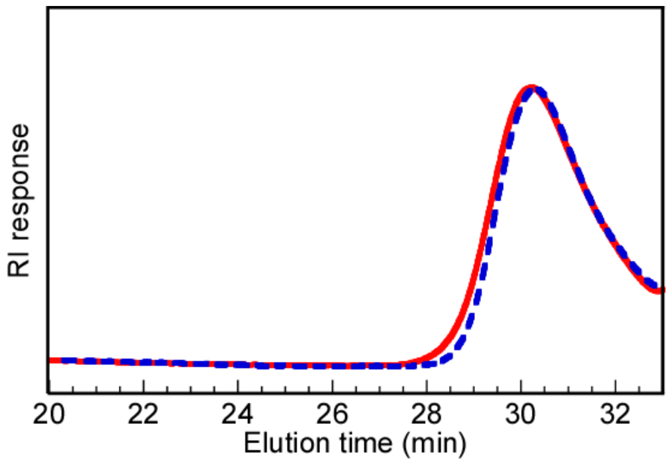

Figure S10. Gel-permeation chromatography (GPC) elution curves of precursor P(MTAC/MPDME43/NBM3) (—) and deprotected P(MTAC/MPA43/NBM3) (----) using acetic acid as the eluent.

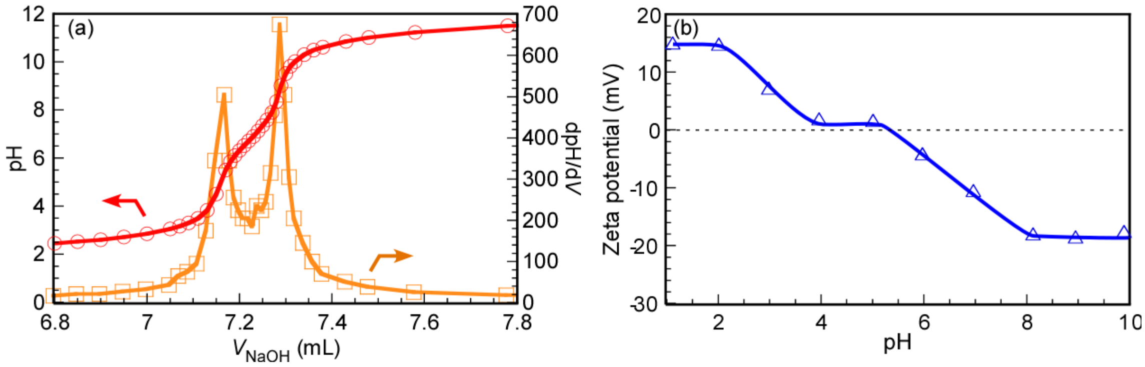

Figure S11. (a) pH (○) and dpH/dV (□) of an aqueous P(MTAC/MPA43/NBM3) solution as a function of the volume of added NaOH solution (V_{NaOH}). (b) Zeta potential of P(MTAC/MPA43/NBM3) as a function of pH in 0.01 M NaCl aqueous solution.

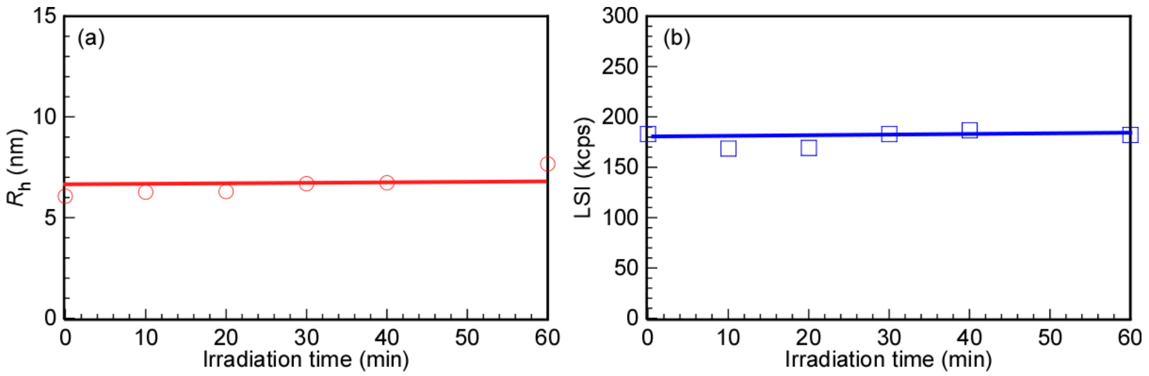

Figure S12. (a) Hydrodynamic radius (R_h) and (b) light scattering intensity (LSI) of P(MTAC/MPA$_{43}$/NBM$_3$) at C_p = 1.0 g/L in acetate buffer at pH 5.0 as a function of photo-irradiation time.

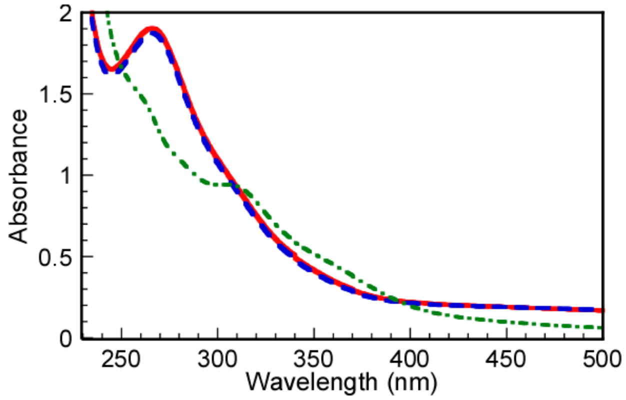

Figure S13. UV-vis absorption spectra of P(MTAC/MPA$_{43}$/NBM$_3$)/PAMPS at f^+ = 0.5 in acetate buffer at pH 5.0 measured immediately after mixing (—), after stirring for 1 h (– –), and after irradiation for 1 h (·—·—).

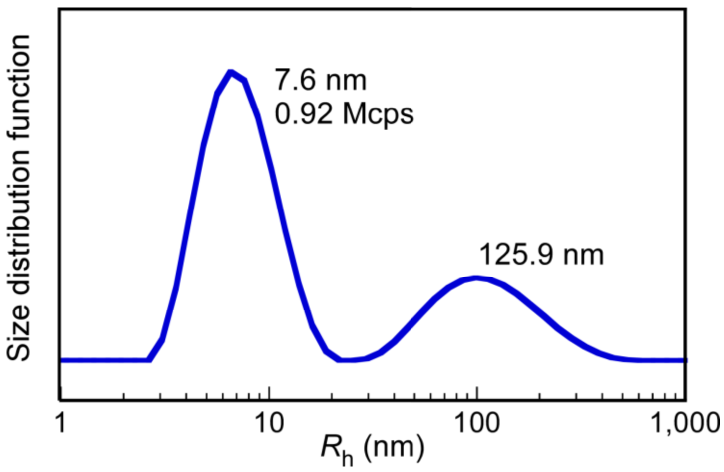

Figure S14. Intensity-weighted hydrodynamic radius (R_h) distributions of P(MTAC/MPA43/NBM3)/PAMPS at f^+ = 0.5 in acetate buffer at pH 5.0 after photoirradiation for 1 h.

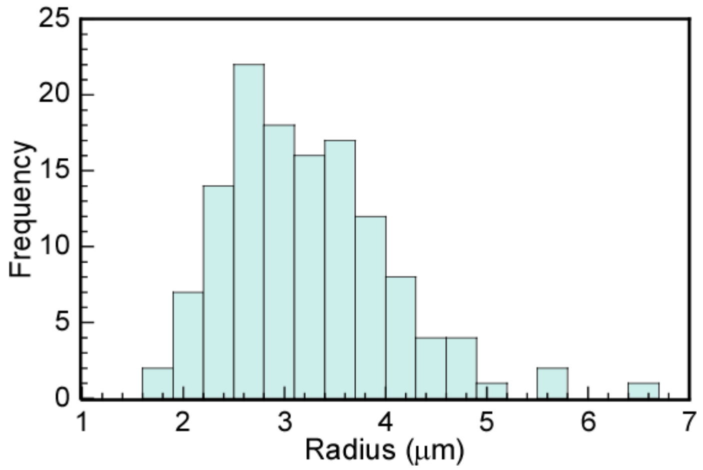

Figure S15. Statistical image analysis of the optical microscopy data for P(MTAC/MPA43/NBM3)/PAMPS at f^+ = 0.5 in acetate buffer at pH 5.0. Distribution of droplet radius before photoirradiation.
